# TMPRSS2:ERG gene aberrations may provide insight into pT stage in prostate cancer

**DOI:** 10.1186/s12894-016-0160-8

**Published:** 2016-07-04

**Authors:** Zoran Krstanoski, Nadja Kokalj Vokac, Andreja Zagorac, Boris Pospihalj, Miha Munda, Saso Dzeroski, Rastko Golouh

**Affiliations:** Department of Urology, General Hospital Slovenj Gradec, Gosposvetska 1, 2380 Slovenj Gradec, Slovenia; Laboratory of Medical Genetics, University Medical Centre Maribor, Maribor, Slovenia; Division of Gynecology, Department of Gynecological Pathology and Cytology, University Medical Center Ljubljana, Ljubljana, Slovenia; Institute for Anatomy, Histology and Embryology, Medical Faculty, University of Maribor, Maribor, Slovenia; Jozef Stefan Institute, Ljubljana, Slovenia; Institute of Pathology, Medical Faculty University of Maribor, Maribor, Slovenia

**Keywords:** FISH, Predicting pT stage, Radical prostatectomy, Prostate cancer, *TMPRSS2:ERG* fusion

## Abstract

**Background:**

*TMPRSS2:ERG* gene aberration may be a novel marker that improves risk stratification of prostate cancer before definitive cancer therapy, but studies have been inconclusive.

**Methods:**

The study cohort consisted of 202 operable prostate cancer Slovenian patients who underwent laparoscopic radical prostatectomy. We retrospectively constructed tissue microarrays of their prostatic specimens for fluorescence in situ hybridization, with appropriate signals obtained in 148 patients for subsequent statistical analyses.

**Results:**

The following genetic aberrations were found: *TMPRSS2:ERG* fusion, *TMPRSS2* split (a non-*ERG* translocation) and *ERG* split (an *ERG* translocation without involvement of *TMPRSS2*). *TMPRSS2:ERG* gene fusion happened in 63 patients (42 %), *TMPRSS2* split in 12 patients and *ERG* split in 8 patients. Association was tested between *TMPRSS2:ERG* gene fusion and several clinicopathological variables, i.e., pT stage, extended lymph node dissection status, and Gleason score, correcting for multiple comparisons. Only the association with pT stage was significant at *p* = 0.05: Of 62 patients with pT3 stage, 34 (55 %) had *TMPRSS2:ERG* gene fusion. In pT3 stage patients, stronger (but not significant) association between eLND status and *TMPRSS2:ERG* gene fusion was detected. We detected *TMPRSS2:ERG* gene fusion in 64 % of the pT3 stage patients where we did not perform an extended lymph node dissection.

**Conclusions:**

Our results indicate that it is possible to predict pT3 stage at final histology from *TMPRSS2:ERG* gene fusion at initial core needle biopsy. FISH determination of *TMPRSS2:ERG* gene fusion may be particularly useful for patients scheduled to undergo a radical prostatectomy in order to improve oncological and functional results.

## Background

Prostate cancer (PCa) is the most common cancer in males and one of the major leading causes of morbidity and mortality. The incidence is higher in Western Europe (>200 per 100,000) than in Eastern Europe [1]. With the widespread use of serum prostatic specific antigen (PSA) screening, almost 90 % of PCa cases can be diagnosed. On the other hand, screening is associated with overdiagnosis and overtreatment with an impact on the patient’s quality of life [2, 3]. Whether underestimated clinically localized cancers should be treated, and if treated, how aggressively they should be treated, remains an important management dilemma. The clinical stage, Gleason grade and the serum PSA levels are used for prognosis and treatment stratification at the time of diagnosis. However, these indicators do not always accurately predict a clinical outcome on an individual patient basis [4]. Thus, more specific diagnostic modalities, prognostic indicators of progression and a better understanding of PCa biology are high priorities in PCa research [5].

The identification of the common *TMPRSS2:ERG* gene fusion in PCa could enable us to detect the disease in an earlier stage and also make it possible to design the proper therapy for each patient. With that we could predict disease outcomes [5, 6], more easily. In this study we examined potential associations between *TMPRSS2:ERG* gene fusion and clinicopathological characteristics with the aim of helping predict the cancer outcome.

## Methods

### Study population

The study cohort consisted of 202 operable PCa patients who underwent laparoscopic radical prostatectomy at the General Hospital Slovenj Gradec in Slovenia, but only 148 patients yielded appropriate signals for futher analysis. Patients were operated in the period between January 2010 and July 2011. Conventional clinicopathological data were evaluated. The mean age of the patients at the time of diagnosis was 64 years (range 47–78) years. The mean value of PSA before operation (Op) was 8.7 ng/ml (range 0.1–110). Extended lymph node dissection (eLND) was performed in 26 patients (13 %), 8 of which had positive lymph nodes (N1) and 18 had no tumor metastasis (N0). The median follow-up time of patients was 36 months. Fifteen patients experienced a biochemical recurrence and underwent hormonal therapy. Up until July 2015, none of these patients had died.

### Generation of tissue microarrays (TMA)

The original haematoxylin and eosin (H&E) slides and paraffin-embedded tumour tissues were retrieved from the archives of the Department of Pathology, General Hospital Slovenj Gradec. H&Estained slides of tumour tissue were reviewed by two pathologists to identify representative tumour regions without necrosis or prostatic intraepithelial neoplasia. Three tissue cylinders with a diameter of 0.6 mm were obtained for each corresponding tumour block and arrayed into a recipient new paraffin block using the tissue chip microarrayer (Beecher Instruments, Silver Spring, MD). Thirty-nine recipient tissue blocks were constructed. The blocks were subsequently cut into 2_3 μm sections and fixed on silanized glass slides (Knittel Glaeser, Germany) to support adhesion of the tissue samples for subsequent fluorescence in situ hybridization (FISH) analysis.

### Interphase

Interphase FISH was performed using the ZytoLight® SPEC *ERG/TMPRSS2* TriChech™ DNA Probe (ZytoVision GmbH, Bremerhaven, Germany), designed to detect deletions between the *ERG* and *TMPRSS2* genes at 21q22 and other translocations affecting either of these genes. FISH was performed on pretreated slides (that had undergone dewaxing, proteolysis, and post-fixation) using the Vysis Paraffin Pretreatment Reagent Kit (Abbott Molecular Inc., Des Plaines, IL, USA), following the manufacturer's instructions. Slides were denatured in 70 % formamide for 10 min at 73 °C, and the probe was denatured for 10 min at 75 °C. The probe was applied to each slide and the slide was covered by a 2--22 mm plastic coverslip and hybridized overnight in a moist chamber at 37 °C. After 16 h, the coverslips were gently removed, and slides were washed in 0.4 × saline sodium citrate (SSC) 0.05 % Tween for 2 min at 73 °C and 2 × SSC 0.05 % Tween for 2--60 s at RT. The cells were counterstained with 4',6-diamidino-2-phenylindole (DAPI) and embedded in an antifade solution. Images were acquired using a Zeiss Axioplan 2 microscope (Goettingen, Germany) equipped with Chroma optical filters (Chroma Technologies, Brattleboro, VT). The FISH results were evaluated by two independent screeners using a 63× objective. In normal interphase nuclei, two red/green/blue fusion signals were expected, representing two normal (non-rearranged) 21q22.13-q22.3 loci.

### Statistical analysis

We tested significance of the association between several nominal clinicopathological variables (i.e., pT stage, eNLD status, Gleason score) and *TMPRSS2:ERG* fusion. For this purpose, we used Fisher’s exact test for two-by-two contingency tables, as well as its Freeman-Halton extension for two-rows by three-columns contingency tables, as appropriate. The significance level was set at 0.05. To correct for the multiple tests, we used Bonferroni correction: Since we test a total of 4 hypotheses (the association of eNLD and *TMPRSS2:ERG* fusion on the subpopulation of pT3 stage patients), the corrected significance level for the individual hypotheses is set to 0.0125. For a significance level of 0.1, the corrected significance level for the individual hypotheses would be 0.025.

## Results

**Fig. 1:**
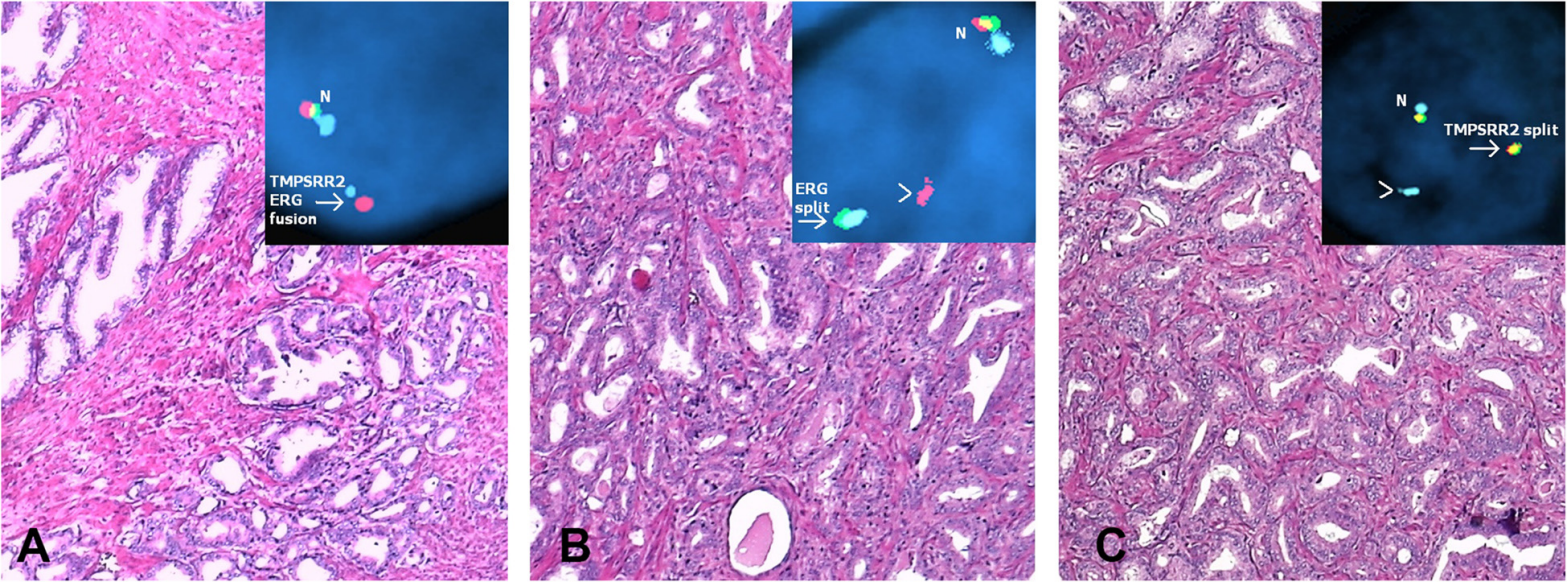
Three cases of invasive adenocarcinoma of the prostate (H&E, 20×) with corresponding FISH staining. Arrow heads show gene aberration (split and fusion) and N shows normal signal. **a** One normal red/green/blue fusion signal and *TMPRSS2:ERG* fusion as indicated by one red/blue fusion signal with loss of one green signal. **b** One normal red/green/blue fusion signal and an *ERG* split as indicated by one green/blue fusion signal with the separated red signal. **c** One normal red/green/blue fusion signal and a *TMPRSS2* split as indicated by one red/green fusion signal with separated blue signal

We performed FISH analyses on 202 operable PCa patients. The sample was classified as aberrant if a staining pattern other than two red/green/blue fusion signals was detected in at least 20 cells in the tumor. *TMPRSS2:ERG* fusion, in wich the 21q22 locus is affected by a 21q22.2 deletion, was indicated as one red/blue fusion signal and the loss of one green signal (Fig. 1a). An *ERG* split without involvement of *TMPRSS2* was indicated by a separated red signal and green/blue fusion signal (Fig. 1b). A *TMPRRS2* split was indicated by a separated blue signal and red/green fusion signal (Fig. 1c). Tumor samples with very weak signals or lack of signals were considered to provide insufficient results and were not considered for further analysis. A resulting 148 patients were considered appropriate for subsequent statistical analysis.

The following genetic aberrations were found in the samples of the 148 patients: *TMPRSS2:ERG* fusion, *TMPRSS2 split* (a non-*ERG* translocation) and *ERG* split (an *ERG* translocation without involvement of *TMPRSS2*). Samples from 79 patients (53 %) had gene aberrations, while no aberrations were detected in 69 patients (47 %). We found *TMPRSS2:ERG* gene fusion in 63 patients (42 %), *TMPRSS2* split in 12 patients and *ERG* split in 8 patients. One patient had both *TMPRSS2:ERG* gene fusion and a *TMPRSS2* split. In three patients, *TMRRSS2:ERG* gene fusion and an *ERG* split were detected.

Table 1 summarizes the clinicopathological characteristics of the 148 patients included in the study. Averages (and counts) are given for all patients, as well as for the groups with/without aberrations and with/without *TMPRSS2:ERG* gene fusion. We tested for significant differences between *TMPRSS2:ERG* gene fusion positive and fusion negative cases in terms of pT stage, N stage and GS before a surgery (Tables 2, 3, and 4).

**Table 1:** A summary of the clinico-pathological data of the 148 patients: Overall, with and without gene aberrations and with/without *TMPRSS2:ERG* fusion

	All Patients	Patients without gene aberrations	Patients with gene aberrations	Patients with fusion *TMPRSS2:ERG*	Patients with no fusion *TMPRSS2:ERG*
	148	69	79	63	85
pT stage					
pT2a	9	5	4	3	6
pT2b	12	7	5	4	8
pT2c	65	34	31	22	43
pT3a	49	20	29	26	23
pT3b	13	3	10	8	5
AGE (years)	64.37 (47–78)	64.50 (47–78)	64.24 (49–76)	64.27 (49–76)	64.44 (47–78)
N stage					
eLND	122	57	65	53	69
pN0	18	8	10	6	12
pN1	8	4	4	4	4
PSA before OP (ng/ml)	8.95 (0.1–110)	9.39 (0.1–110)	8.56 (0.71–43.2)	8.37 (0.71–43.2)	9.38 (0.1–110)
GS before OP (N°)					
6	62	35	27	21	41
7	78	32	46	38	40
≥8	8	2	6	4	4
PSA 1 month after OP ng/ml	0.26 (0.0–20)	0.42 (0.0–20)	0.11 (0.00–2.8)	0.14 (0.00–2.8)	0.35 (0.001–20)
GS after OP (N°)					
6	47	28	19	15	32
7	88	36	52	42	46
≥8	13	5	8	6	7

**Table 2:** Association of pT (stage) with gene fusion: Contingency table. The *p*-value obtained by the Fisher’s exact test for two-by-two contingency tables is 0.01. After Bonferroni correction for testing 4 hypotheses, this is still significant at the 0.05 level

	*TMPRSS2-ERG*	No fusion	Total
pT2	29	57	86
pT3	34	28	62
Total	63	85	148

**Table 3:** Association of Gleason score (before operation) with gene fusion: Contingency table. The *p*-value obtained by the Freeman-Halton extension of Fisher’s exact test for two-by-three contingency tables is 0.19. After Bonferroni correction for testing 4 hypotheses, this is not significant at the 0.05 level

Gleason score	*TMPRSS2-ERG*	No fusion	Total
6	21	41	62
7	38	40	78
≥8	4	4	8
Total	63	85	148

**Table 4:** Association of N stage (eLND status) with gene fusion: Contingency table. The *p*-value obtained by the Freeman-Halton extension of Fisher’s exact test for two-by-three contingency tables is 0.66. After Bonferroni correction for testing 4 hypotheses, this is not significant at the 0.05 level

	*TMPRSS2-ERG*	No fusion	Total
eLND	53	69	122
pN0	6	12	18
pN1	4	4	8
Total	63	85	148

The association between *TMPRSS2:ERG* fusion and pT stage (with values divided into two groups, pT2 and pT3) was significant. The exact *p*-value obtained by Fisher’s exact test was 0.01, which after the Bonferroni correction still makes the association significant at the 0.05 level. From 62 patients with pT3 stage, *TMPRSS2:ERG* gene fusion was detected in 34 (55 %) (Table 2).

The association between *TMPRSS2:ERG* fusion and Gleason score before operation was not significant. The exact *p*-value was 0.19. The contingency table is given in Table 3. This was obtained by the Freeman-Halton extension of Fisher’s exact test.

The association between *TMPRSS2:ERG* fusion and N stage (eLND status) was examined next, considering the subgroups of eLND, pN0 and pN1 patients. In the entire population of 148 patients, the association was very weak and thus not significant. The exact *p*-value was 0.66 (Table 4). Again, the *p*-value was obtained by the Freeman-Halton extension of Fisher’s exact test.

For further statistical analysis, we considered only the patients with pT3 stage. We re-examined the association between *TMPRSS2:ERG* fusion and N stage in this subpopulation. Here, the association was much stronger (exact *p*-value of 0.02), but not significant at the 0.05 level due to the Bonferroni correction. It is only significant at the weaker 0.1 level. In the group of patients on which we did not perform eLND, we detected *TMPRSS2:ERG* gene fusion in 25 patients (64 %).

## Discussion

Many new biomarkers have been recently tested to enhance the accuracy of diagnosis, prediction of stage, estimation of metastatic potential and biochemical recurrence of PCa. So far, only a few have shown positive results and promising practical use in everyday practice. One of the most promising biomarkers is *TMPRSS2:ERG* gene fusion. This fusion is important not only for its high prevalence (or combining with other biomarkers), sensitivity and specificity in early diagnosis of PCa [7, 8], but also in predicting the stage [9–11], aggressiveness [9, 12] and metastatic potential of the tumor [11, 13].

In our study of 148 patients, we observed a prevalence of gene fusion in 42 %, which is similar to published data reporting prevalence ranges of 44–50 % [14–16]. Futhermore, we found no differences between fusion positive and negative cases in relation to age, PSA and GS. This is in concordance with the study by Magi-Galluzzi et al. that included 42 Caucasians, 64 African-Americans, and 44 Japanese patients who underwent radical prostatectomy [17]. *TMPRSS2:ERG* gene fusion correlated with ethnicity (*p* = 0.03) and marginally correlated with pathologic stage (*p* = 0.06), but did not correlate with other clinicopathologic parameters, such as age, preoperative PSA levels, and GS. Pettersson et al. [18] did not find any statistical significance betveen TMPRSS2:ERG gene fusion and GS (either low grade or high grade). Their cohort study includes 1180 patients after radical prostatectomy. with 694 patients showing GS ≤ 7 and 355 with positive gene rearrangement; a total of 486 patients had poorly differentiated prostate carcinoma GS ≥8 and 229 patients had gene fusion (*p* = 0.58). Similarly, Perner et al. [11] did not observe any significant associations between GS and *TMPRSS2:ERG* status in their study of 118 patients.

Gopalan et al. [19] reported different results. The authors found a statistically significant correlation between gene fusion and low GS (*p* = 0.02). Gene fusion was found in 71 patients (32 %) with GS < 7, 118 patients (54 %) with GS = 7 and 16 patients (7 %) with GS > 7. Seventy-four patients (24 %) showed GS < 7, while 182 patients (60 %) with GS = 7 and 40 patients (13 %) with GS > 7 had no gene fusion.

In the study of Darnel et al. [20] of 196 patients, the authors found a statistically significant correlation between *TMPRSS2:ERG* gene fusion and lower primary Gleason pattern. Gene fusion was detected in 42 % of patients with primary Gleason pattern 3 and 27 % in primary Gleason pattern 4 (*p* = 0.014).

Demichelis et al. [12] showed a statistical significance between *TMPRSS2:ERG* gene fusion and higher GS (*p* = 0.01). Similar connections between gene fusion and GS ≥ 8 were shown in study by Font-Tello et al. [21].

The results of our study show a significant association between *TMPRSS2:ERG* gene fusion and pT stage. Perner S. et al. [11] reported high percentage of *TMPRSS2:ERG* rearrangements in patients with pT3 stage, i.e., 50/91 patients (55 %) and *p* = 0.03. Mehra et al. [10] reported similar findings for pT2b, but in the other direction. The authors found a statistically significant association between *TMPRSS2* gene rearrangement and the presence of advanced pathologic tumour stage (*p* = 0.04), defining advanced stage as pT2b. In their study, a total of 24 out of 37 (65 %) patients with positive *TMPRSS2* rearrangements had pathologic tumour stage ≤ pT2b. Font-Tello et al. [21] analyzed the mRNA levels of *TMPRSS2-ERG, ERG, PTEN*, and AR (*n* = 83), as well as *ERG* immunostaining (*n* = 78) in a series of prostate tumors. They found *TMPRSS2:ERG* gene fusion in 57 patients and it was associated with stage T3-T4 tumors. Saramäki et al. [22] did not find correlation between gene fusion and T3 stage (*p* = 1.0).

In our study *TMPRSS2:ERG* gene fusion was twice as frequent in the group of pT3 patients who did not undergo eLND compared with the group of pT3 patients who did undergo eLND. This finding indicates that we probably underestimate the group of patients with classical prognostic factors that characterise these patients as low or intermediate risk of PCa.

As we only had five patients with* ERG* split alone and three patients with both *ERG* split and *TMPRSS2:ERG* gene fusion, which was only 10 % of all gene rearrangements, the number of this subgroup was too small for statistical analyis.

Methodological differences in the patient cohorts could lead to these discrepancies. Some recent studies have shown that genetics diferences in prostate cancer among interracial groups can also be a reason for these discrepancies [23].

This study had several limitations. The study is retrospective in nature and prone to selection and collection bias. In addition, the sample size was fairly low, limiting subgroup data analyses. Therefore, all data should be confirmed in large possible prospective cohorts.

If confirmed in larger studies gene rearrangements on biopsies or postoperative specimens could be useful adjunct to clinical routine markers. In addition, it may be possible to detect these rearrangements in urine samples, eliminating the need for invasive specimen collection.

## Conclusions

Based on our results, we expect that more than half of the patients with *TMPRSS2:ERG* gene fusion at their initial core needle biopsy will have a pT3 stage at their final histology in more than one half of the cases. This is especially important for patients with preoperative PSA and GS values indicating a low or intermediate risk of PCa. For one half of them, their preoperative clinical stage is probably underestimated. If, in these patients, we expect a pT3 stage (at their final histology), this could help us to achieve a lower percentage of positive section margins, as published (33.5–66 %) [24]. Namely, in patients with a clinical pT3 stage, we expect higher prevalence of positive lymph nodes (7.9–49 %) [24]. Following the results of our present study, we have to indicate eLND in patients with confirmed gene fusion, even if they have “false” low risk PCa (as indicated by their preoperative PSA and GS values). To conclude, *TMPRSS2:ERG* gene fusion at the initial core needle biopsy may be associated with pT3 stage and may therefore represent a biomarker for clinical routine. FISH determination of *TMPRSS2:ERG* gene fusion may be particularly useful for patients scheduled to undergo a nerve-sparing procedure in order to improve oncological and functional results. However, it is difficult to support the theory that patients with gene fusion will have worse prognosis.

## Abbreviations

eLND, extended lymph node dissection; FISH, fluorescence in situ hybridization; GS, Gleason score; N0, negative lymph node; N1, positive lymph node; Op, operation; PCa, prostate cancer; PSA, prostatic specific antigen; TMA, tissue microarrays
